# Bioactive silicon-based dental gel associated with a calcium booster for dentin hypersensitivity treatment: A randomized clinical study

**DOI:** 10.4317/jced.62205

**Published:** 2024-12-01

**Authors:** Leandro Araujo Fernandes, Ana Luiza Marques Reis, Marcela de Cassia dos Reis, Thamires Mazzola, Joao Vitor da Cruz Pegoraro, Sebastiao Orestes Pereira Neto, Daniela Coelho de Lima, Paulo Henrique Perlatti D’Alpino

**Affiliations:** 1 School of Dentistry, Universidade Federal de Alfenas (UNIFALMG), Alfenas, Minas Gerais, Brazil; 2 Triplet Biotechnology Solutions, Inc., Bauru, SP, Brazil; 3 São Paulo State University (UNESP), School of Sciences, Bauru, SP, Brazil

## Abstract

**Background:**

Dentin hypersensitivity affects a significant portion of the world’s population, causing pain and negatively impacting oral health and quality of life This clinical study investigated the effectiveness of a desensitizing fluoride silicon-rich dental gel associated with a calcium booster.

**Material and Methods:**

In this single-blind, randomized, parallel-group clinical trial, forty-eight volunteers diagnosed with dentin hypersensitivity and qualified to participate were randomized into 2 groups: (1) fluoride silicon-rich dental gel (REFX Technology)/calcium booster (Si/Ca protocol); (2) Pumice stone diluted in saline. A draw was carried out between the right and left sides of the patients so that either a prophylaxis with the dental gel associated with calcium or with fine pumice stone diluted in saline was carried out. An evaporative test was used to check for dentin hypersensitivity in the subjects before and immediately after treatment and scored using the Schiff scale, rated 0 to 3. Data were statistically analyzed (ANOVA/Tukey test, α=5%).

**Results:**

The treatment with Si/Ca protocol drastically reduced dentin hypersensitivity (from 2.58 to 0.67) immediately after the use. In the control group (Pumice stone), similar results were observed before and after treatment (2.67 and 2.82, respectively).

**Conclusions:**

The Si/Ca protocol was fast and effective in reducing pain caused by dentin hypersensitivity.

** Key words:**Clinical trial, dentin hypersensitivity, desensitizing, toothpaste.

## Introduction

Dentin hypersensitivity (DH) is a brief, acute, localized, and temporary discomfort induced by dentin exposure (1,2). DH affects a significant portion of the world’s population, causing pain and negatively impacting oral health and quality of life (3,4). Its frequency in the adult population ranges from 3% to 57%, with a maximum of 98% in individuals with periodontal disease. In most cases, women are impacted by DH (5-7). In general, the treatment is based on two main strategies: preventing fluid movement by obliterating the dentinal tubules and/or blocking the nerve signals to the pulpal receptors, which disrupts the response to painful stimuli (2). Finding a treatment that is quick, effective, and eliminates the painful sensation of hypersensitivity while preventing recurrence is the major challenge facing dentistry today (2).

Products containing silicon-based technologies have been used in the management of DH, providing strong evidence that these technologies can reduce dentin hypersensitivity and promote enamel-dentin repair. In this sense, dentifrices containing silicon, silica or silicate compounds can act as dentinal tubule obliterators, one of the pain control mechanisms (8-12).

A previous case report (7) described a clinical protocol in which the authors proposed a dental prophylaxis with a silicon desensitizing toothpaste associated with calcium booster in order to diminish DH. The tooth mineral gain is facilitated by the association with boosters or supplements, even when associated with fluoride in low-F applications (13). For comparative reasons, the same procedure was performed in the contralateral teeth using pumice stone diluted in saline. In both areas, the prophylaxis was performed with a Robson brush for 10 s in each exposed area. The authors used an evaporative test to analyze the pain discomfort using the Schiff Air Index, with scores ranging from 0 to 3, by applying a 5-second burst of air to the exposed dentin surface before and after the prophylaxis. The patient reported pain before the treatment and interruption of the stimulus before and after prophylaxis with the dental gel associated with a calcium booster (before SAI: 3; after: SAI 1). Conversely, no pain relief was observed when the prophylaxis was performed with pumice stone (SAI 3 in both evaluation times).

In that study, the authors used a dental gel containing REFIX technology associated with a calcium booster (7). According to another previous study, this association favors an ionic alteration at the dental tissues, which allows the creation of fluoridated apatite and the formation of a silicate layer, which is also formed deeper into the enamel tissues and open dentin tubules (14). As a consequence, there is a decrease in hydroxyapatite solubility as well as a decrease in dentin fluid flow, protecting against the consequences of dentin hypersensitivity (5). The calcium supplement provided by the booster improves the availability of calcium in the oral environment and accelerates the enamel remineralization process. It has been previously reported an *in vitro* study that the association fluoride silicon-rich toothpaste/calcium booster promotes the regeneration of the dental tissues, effectively remineralizing the enamel structure and occluding the dentin tubules (12). Bioactive agents derived from silicon-based systems, such as REFIX technology, have been used in dental gels to promote tooth remineralization/regeneration, with a focus on tooth sensitivity treatment (6,11,15,16).

The objective of the present clinical study was to investigate the effectiveness of the clinical protocol consisting of a dental prophylaxis performed with the association fluoride silicon-rich dental gel/calcium booster (Si/Ca protocol) in order to reduce the immediate dentin hypersensitivity reported by voluntary subjects. The research hypothesis was that using this clinical protocol will help to reduce/control the immediate discomfort experienced by participants with dentin hypersensitivity.

## Material and Methods

-Experimental design

This study was a single-blind, randomized, parallel-group clinical study to evaluate the efficacy of a regenerative dental gel associated with a calcium booster to relieve the symptoms of dentin hypersensitivity. The primary outcome to be examined was the reduction of the immediate dentin hypersensitivity from baseline in response to the proposed treatment using an evaporative test (Schiff scale) (17). The study was conducted at the School of Dentistry of the Universidade Federal de Alfenas (UNIFALMG, Brazil). The factors under analysis were I. Treatment, in 2 levels: 1) Dental gel (Regenerador Sensitive DentalClean) associated with calcium booster; 2) Pumice stone prophylaxis; II. Evaluation time, in 2 levels: 1) baseline: before the treatment; 2) immediately after the treatment.

The sample size was calculated using Sealed Envelope Ltd. (18) considering a significance level (alpha) of 5% and a power (1-beta) of 80%, and at least 48 teeth per group were included. Even with a possibility of a dropout rate of 25%, a minimum of 48 subjects were included in this study.

-Ethical Considerations

The present study was conducted in accordance with the Declaration of Helsinki (1964) and its later amendments and registered at Clinicaltrial.gov (RBR-6jts634). Before enrolling in the study, all participants received accurate details about the clinical trial and then signed an informed written permission form.

-Patient selection

**Figure 1:**
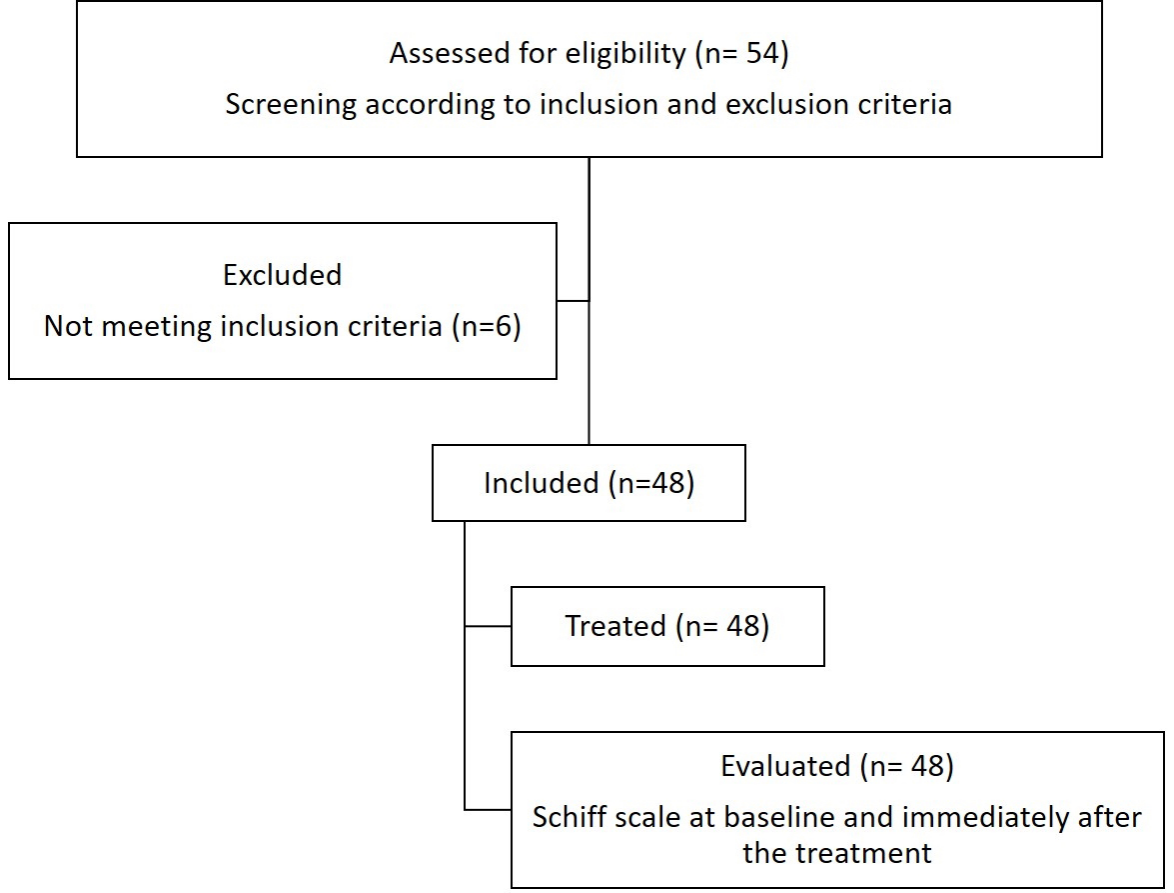
CONSORT diagram of participant flow for this clinical trial.

This clinical trial followed the CONSORT statement. For that, 54 patients, both male and female, presenting dentin hypersensitivity were evaluated for eligibility (Fig. 1). The inclusion criteria were subjects between 18 and 65 years old; in good health, with no history of allergies to dentifrice ingredients (essentially preservatives and flavorings); classified, according to the American Society of Anesthesiology (ASA), in group I (healthy, without systemic alterations or making continuous use of medication). Subjects were selected with at least two contralateral teeth with exposed root surface due to abrasion, abfraction, erosion, or gingival recession, which provoked a painful reaction to an air blasting stimulus, with a minimum baseline score of 2 (17). Figure 1 displays the CONSORT diagram of participant flow for this clinical trial.

Patients presenting physiological changes who were not classified as ASA I (pregnant women, patients with chronic diseases, patients with infectious foci in the oral cavity); with orthodontic appliances, moderate or advanced active periodontal disease, soft or hard oral tissue tumors, and pregnant women were excluded from the study. Subjects who used analgesics or other medications that could potentially mask pain sensation (such as antihistamines, sedatives, anticonvulsants, antidepressants, antiinflammatory drugs, tranquilizers, or daily analgesics), as were those who routinely used desensitizing toothpaste or any other desensitizing agent were also excluded. In the same manner, subjects reporting hypersensitivity in teeth with enamel cracks or flaws, cavitated caries, fractures, mobility, extensive or poor restorations, prosthetic crowns or veneers, or pulp involvement were also excluded from the study. Three of the 54 patients initially chosen were excluded for not meeting the inclusion criteria; 48 subjects were included in the study (Fig. 1).

-Clinical procedures

A draw was carried out between the right and left sides of the patients so that either a prophylaxis with the dental gel associated with calcium or with fine pumice-stone diluted in saline was carried out. The Si/Ca protocol was applied to the buccal surface of teeth after mixing equal parts (1:1). The Si/Ca protocol (REFIX Booster, Rabbit Corp, Londrina, Brazil) consists in a dental gel containing REFIX Technology as known as tube 1 - Crystallizer (Silicon/phosphate gel) and tube 2 - Accelerator (Calcium gel). The diluted pumice stone was applied to the buccal surfaces of the teeth on the contralateral side. Both treatments were performed for 10 seconds with the aid of Robson brush. Then, a 5-second burst of air from a triple 3-way syringe handpiece was administered to the exposed dentin surface at a distance of around 0.5 cm to assess discomfort. In the Schiff scale, the participants are scored from 0 to 3: score 0- subject does not respond to stimulus; score 1- subject responds to stimulus, but does not request discontinuation of stimulus; score 2- subject responds to stimulus and requests discontinuation or moves away from stimulus; score 3- subject responds to stimulus, considers it to be painful, and requests discontinuation of the stimulus (17). This scale is filled out by the operator. The pain scores were measured at baseline (before treatment), and then right after following the application of the desensitizing toothpaste. The rate of pain severity following air blasting was observed immediately after the air stimulus.

-Statistical analysis

After analyzing data normality using the Shapiro-Wilk test, the statistical analysis was performed using two-way ANOVA/Tukey’s test at a pre-set of 5%. This test was selected for comparative evaluation of the decrease rate (%) of toothpaste pain after each evaluation period (immediately after brushing).

## Results

The average age of the subjects in this clinical study was 46 years, and 53.84% of the 48 participants were female.  Table 1 shows the mean pain level based on the evaporative test evaluation period. The results of the present study demonstrated that there was no difference in scores of pain before and after the prophylaxis of teeth using pumice stone diluted in saline after stimuli. In the group treated with the Si/Ca protocol, a significant difference was observed after application when comparing the pain scores of the initial and final stimuli (2.58 and 0.67, respectively; *p* < 0.05). Regarding the comparison of the pain scores, comparing the final stimuli between the groups, a significant difference in dentin hypersensitivity was observed when the treatments with the combination fluoride silicon-rich toothpaste/calcium booster and pumice stone were compared (2.82 and 0.67, respectively; *p* < 0.05).

**Figure 2:**
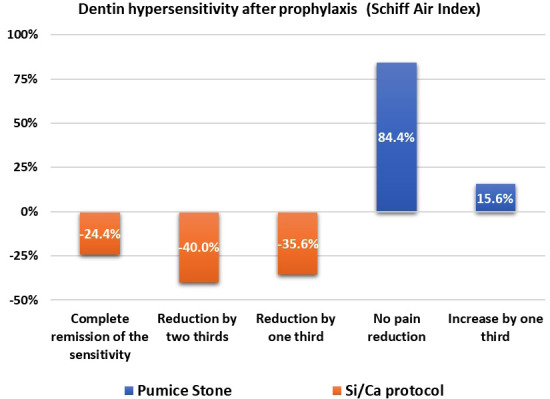
Distribution of the percentual of pain manifestation after treatment according to the Schiff scale.

Figure 2 displays the distribution of the percentual of pain manifestation after treatment according to the Schiff scale. It can be observed that, immediately after the application of the Si/Ca protocol, the evaporative analysis indicated that 24.4% of the teeth obtained complete remission of sensitivity (score 0). Additionally, 40.0% experienced a higher decrease in sensitivity levels (around 70%), and 35.6% experienced one third of pain relief. Conversely, when the teeth were treated with pumice stone diluted in saline, 84.4% of the subjects reported no pain relief, whereas 15.6% reported pain augmentation by one third.

## Discussion

The findings in this study showed pain relief when the Si/Ca protocol was used in a prophylaxis dental protocol. According to  Table 1, a significant reduction in the pain scores was observed after the treatment when compared to that before treatment with the Si/Ca protocol. Results of the present study were corroborated in *in vitro* and *in vivo* studies (5,9-12,15,16,19-21) which demonstrated the efficacy to treat dentin hypersensitivity using silicon-based technologies.

It was also demonstrated that 15.6% of the teeth treated with pumice stone diluted in saline had an increase in one-third of dentin hypersensitivity. In addition, subjects reported no pain reduction in 84.4% of evaluated teeth. On the other hand, in the in the Si/Ca protocol, 24.43% of teeth presented complete pain relief, and 33.3% of teeth had a reduction in pain in two thirds. In 35.60% of teeth, there was pain relief by one third (Fig. 2).

A previous study reported that the Schiff scale presented the best scores for sensitivity and specificity (22). The high values for these two characteristics allow a diagnosis that encompasses the maximum correct results with the fewest false negatives and false positives in relation to the actual diagnosis. This result was expected, considering that the Schiff scale was developed specifically for dentin hypersensitivity assessment (23,24). It is also important to note that the Schiff scale was the only scale applied by the operator; all other scales were self-reported by the participants.

The Si/Ca protocol was developed based on the REFIX technology. consists of an acidified bioactive complex made up of organic molecules, salts, and substances related to phosphates and silicon (11,15,16,19). According to the manufacturer, this relationship promotes the creation of fluoridated apatite and the formation of a silicate layer, which is also formed deeper into the enamel tissues and open dentin tubules. Also, the formation of this layer is accelerated by the combination of compounds containing silicon and calcium (8). This alleged ionic alteration promotes a decrease in hydroxyapatite solubility, a decrease in dentin fluid flow, and an improvement in dentin mechanical characteristics. As a result, this mechanism protects against the consequences of dentin hypersensitivity (5).

In a previous *in vitro* study (16), it was demonstrated in a morphological analysis that a mineral layer formed on the treated enamel surface; the layer had a consistent uniform thickness of ~14 µm. These results were corroborated in another study, which also demonstrated the formation of a silicon-enriched mineral layer on the enamel and dentin surfaces. In the latter, the dentinal tubules were progressively occluded until a complete tubule occlusion occurred after 7 days (11). These results were also corroborated in a previous clinical study, which demonstrated an immediate effect after the first use, where the pain reported by the subjects reduced to mild pain according to the visual analogue scale (VAS), ranging from 0 to 100. After one week of consistent use, the pain score was significantly reduced, with most participants reporting no pain, proving the effectiveness of fluoride silicon-rich toothpaste against dentin hypersensitivity (5).

A previous clinical trial conducted in 53 volunteers assessed the efficacy of a fluoride silicon-rich toothpaste against dentin hypersensitivity (5). The baseline data revealed a mean VAS (100-0) pain index of severe pain (VAS 65). There was an average decrease to mild pain (VAS 25) after the first brushing, with one-third of the group no longer experiencing pain. An average pain-free score (VAS 07) was found after one week of treatment (5).

In another clinical trial, it was investigated and compared the effectiveness of toothpastes containing bioactives to relieve dentin hypersensitivity with that of a commercial desensitizing fluoride silicon-rich toothpaste associated or not with a calcium booster (14). The subjects brushed their teeth with one of the toothpastes, and dentin hypersensitivity was immediately tested using a VAS scale (from 0 to 100). Dentin hypersensitivity was measured after one week and after one month of the subjects continuing to use the toothpaste three times per day. In the evaporative test, Sensodyne Protect & Repair and Regenerador Sensitive, associated with the calcium booster, exhibited faster and more effective results in reducing pain caused by dentin hypersensitivity, even after the first use. The bioactive toothpastes reduced, to a different extent, the dentin hypersensitivity reported by the volunteers. Sensodyne Repair & Relief and Regenerador Sensitive, associated or not with a calcium booster, presented faster and more effective results in reducing pain caused by dentin hypersensitivity (14).

Another clinical trial, it was demonstrated the benefits of both fluoride silicon-rich toothpaste (Regenerador Sensitive) and NovaMin-based toothpaste to occlude dentin tubules and consequent reducing dentin hypersensitivity in 18 periodontal patients using the VAS and Schiff pain scales (6). There were no significant differences between the technologies after eight weeks of treatment. It was observed an average reduction from severe pain (VAS 64) to mild pain (VAS 21) after using both toothpastes. Similarly, SAI pain scale revealed a mean reduction in dentin hypersensitivity when both products used (SAI 2.26-0.56) (6).

Results of the present clinical study proposed a new protocol in which the treatment with The Si/Ca protocol significantly reduced dentin hypersensitivity immediately after the use. The pain, according to the Schiff scale, varied from 2.58 to 0.67 after the treatment, which represents an important gain in terms of quality of life, especially considering the patients with extensive exposed dentin areas after periodontal treatment. A possible limitation of the study could be associated with continued use and assessment of the maintenance of sensitivity remission. In this manner, more clinical studies are necessary to evaluate the long-term results of this clinical protocol and eventually the need for recurrent prophylaxis and/or other application forms to maintain the pain relief.

## Conclusions

The results of this clinical study demonstrated that the dental prophylaxis performed with combination of fluoride silicon-rich dental gel and calcium booster was beneficial in reducing the immediate dentin hypersensitivity reported by the participants. Based on these findings, this therapeutic approach is a potential option for reducing pain and discomfort immediately following periodontal treatment.

## Figures and Tables

**Table 1:** Schiff scale values before and after the prophylaxis protocols.

Prophylaxis Protocol	Schiff Air Index (SAI)	
	Before treatment (baseline)	After treatment
Pumice Stone diluted with saline	2.67 (0.47)	2.82 (0.38)^ a^
Dental gel associated with Calcium	2.58 (0.50)^ b^	0.67 (0.47) ^a,b^

n = 48 Same lower-case letter “b” for row: significant (*p*<0,05). Same lower-case letter “a” for column: significant (*p*<0.05)

## Data Availability

The datasets used and/or analyzed during the current study are available from the corresponding author.
